# Total Psoas Muscle Area as a Marker for Sarcopenia Is Related to Outcome in Children With Neuroblastoma

**DOI:** 10.3389/fsurg.2021.718184

**Published:** 2021-08-19

**Authors:** Annika Ritz, Alexandra Froeba-Pohl, Julian Kolorz, Victor Vigodski, Jochen Hubertus, Julia Ley-Zaporozhan, Dietrich von Schweinitz, Beate Häberle, Irene Schmid, Roland Kappler, Eberhard Lurz, Michael Berger

**Affiliations:** ^1^Department of Pediatric Surgery, Dr. von Hauner Children's Hospital, Ludwig-Maximilians-University Munich, Munich, Germany; ^2^Department of Radiology, Pediatric Radiology, Ludwig-Maximilians-University Munich, Munich, Germany; ^3^Department of Pediatrics, Division of Hematology and Oncology, Dr. von Hauner Children's Hospital, Ludwig-Maximilians-University Munich, Munich, Germany; ^4^Department of Pediatrics, Division of Gastroenterology and Hepatology, Dr. von Hauner Children's Hospital, Ludwig-Maximilians-University Munich, Munich, Germany; ^5^Department of General, Abdominal, and Transplant Surgery, Essen University Hospital, Essen, Germany

**Keywords:** psoas muscle surface area, sarcopenia, neuroblastoma, children, biomarker, nutrition

## Abstract

**Background:** Sarcopenia describes a generalized loss of skeletal muscle mass, strength, or function. Determined by measuring the total psoas muscle area (tPMA) on cross-sectional imaging, sarcopenia is an independent marker for poor post-surgical outcomes in adults and children. Children with cancer are at high risk for sarcopenia due to immobility, chemotherapy, and cachexia. We hypothesize that sarcopenic children with neuroblastoma are at higher risk for poor post-operative outcomes.

**Patients and Methods:** Retrospective analysis of children with neuroblastoma ages 1–15 years who were treated at our hospital from 2008 to 2016 with follow-up through March 2021. Psoas muscle area (PMA) was measured from cross-sectional images, using computed tomography (CT) and magnetic resonance imaging (MRI) scans at lumbar disc levels L3-4 and L4-5. tPMA is the sum of the left and right PMA. Z-scores were calculated using age- and gender-specific reference values. Sarcopenia was defined as a tPMA z-score below −2. A correlation of tPMA z-scores and sarcopenia with clinical variables and outcome was performed.

**Results:** One hundred and sixty-four children with workup for neuroblastoma were identified, and 101 children fulfilled inclusion criteria for further analysis, with a mean age of 3.92 years (SD 2.71 years). Mean tPMA z-score at L4-5 was −2.37 (SD 1.02). Correlation of tPMA z-score at L4-5 with weight-for-age z-score was moderate (*r* = 0.54; 95% CI, 0.38, 0.66). No association between sarcopenia and short-term outcome was observed. Sarcopenia had a sensitivity of 0.82 (95% CI, 0.62–0.93) and a specificity of 0.48 (95% CI 0.36–0.61) in predicting 5-year survival. In a multiple regression analysis, pre-operative sarcopenia, pre-operative chemotherapy in the NB2004 high-risk group, unfavorable tumor histology, and age at diagnosis were associated with 5-year survival after surgery, with hazard ratios of 4.18 (95% CI 1.01–17.26), 2.46 (95% CI 1.02–5.92), 2.39 (95% CI 1.03–5.54), and 1.01 (95% CI 1.00–1.03), respectively.

**Conclusion:** In this study, the majority of children had low tPMA z-scores and sarcopenia was a risk factor for decreased 5-year survival in children with neuroblastoma. Therefore, we suggest measuring the tPMA from pre-surgical cross-sectional imaging as a biomarker for additional risk stratification in children with neuroblastoma.

## Introduction

Sarcopenia is defined as the progressive and generalized loss of skeletal muscle mass, strength and function (performance) with a consequent risk of adverse outcomes (1). Children with malignancies are vulnerable to sarcopenia due to chronic inflammation, reduced physical activity, and a reduced intake and uptake of food (2–8). Validated techniques to measure the loss of skeletal muscle mass include dual-energy x-ray absorptiometry (DXA), bio-electric impedance analysis (BIA), cross-sectional computed tomography (CT) scanning, and magnetic resonance imaging (MRI) (1, 9, 10). Studies have shown that a low total psoas muscle area (tPMA), measured by adding the left and right psoas muscle between intervertebral lumbar disc level L3-L5 on MRI or CT imaging, is an independent risk factor for adverse outcomes in adults undergoing surgery or with chronic illnesses (11–18). A growing body of pediatric research shows that a low skeletal muscle mass on CT and MRI images is associated with poor outcome in children with acute lymphocytic leukemia, appendicitis, end-stage organ failure, hepatoblastoma, and high-risk neuroblastoma (NB) (3, 4, 19–22). Recently, age and gender-specific tPMA reference values for lumbar levels L3-4 and L4-5 have been published, which has simplified the assessment of sarcopenia in children (10).

We hypothesized that sarcopenia is an additional risk factor for poor post-surgical outcome in children with NB. Using age- and gender-specific tPMA reference values, our study aimed to assess whether children with NB were sarcopenic prior to tumor resection and whether sarcopenia was associated with an adverse short- and long-term outcome.

## Materials and Methods

### Study Population and Design

All children between 1 and 15 years of age, who underwent a workup for NB at our institution between October 2008 and January 2016, were included in our retrospective study. Clinical data, including follow-up, was collected until March 2021. Children were excluded due to unavailable pre-operative CT or MRI, low image quality (e.g., artifacts), omitted lumbar levels L3-5 on imaging, no surgical procedure, or vertebral anomalies. Anthropometric data, laboratory data, clinical parameters, and outcome information were retrieved through the hospital's electronic medical record system. The study was approved by the local Research Ethics Committee (Project Number 18-681).

### Psoas Muscle Surface Area Measurements

CT and MRI images were retrieved through the hospital's picture archiving and communication system (PACS) and examined through the Digital Imaging and Communications in Medicine (DICOM) viewer (Syngo V36). The left and right psoas muscle areas were measured in mm^2^ at two lumbar levels (L4-5 and L3-4) on axial view with the provided region of interest (ROI) measurement tool. The sum of both sides was calculated to compute the tPMA (20). T2 weighted images were used for MRI scans. Z-scores were calculated using a dedicated online tool (https://ahrc-apps.shinyapps.io/sarcopenia/) (10). Sarcopenia was defined as a z-score smaller than −2. Measurements were performed by AR, and, in 30% of cases, verified by radiologist and co-author JL-Z.

### Clinical Parameters and Outcome Markers

Clinical parameters included pre-operative height, pre-operative weight, tumor stage according to the International Neuroblastoma Staging System (INSS) (23), tumor localization, tumor histology, MYCN gene amplification (MYCN+) (24), age at diagnosis, and pre-operative chemotherapy. Poorly differentiated neuroblastoma, undifferentiated neuroblastoma, and nodular ganglioneuroblastoma were considered unfavorable histology (25). Weight-for-age (WFA), height-for-age (HFA), and body mass index (BMI) percentiles and z-scores were calculated using the gender- and age-specific WHO anthropometric calculator (WHO Anthro version 3.2.2) (26).

Short-term outcome markers included total post-operative days in the intensive care unit (ICU), total post-operative days in the hospital, and post-operative sepsis with positive blood culture. Long-term outcome included relapse and disease status [complete remission (CR), alive with disease (AWD), progressive disease (PD), death of disease (DOD)].

### Statistical Analysis

Continuous variables are presented as mean with standard deviation (SD) or median with interquartile range (IQR). Categorical variables are presented as percentages or proportions. Comparison between continuous data was done with Student *t*-test, Mann-Whitney *U*-test, Ordinary one-way ANOVA, and Kruskal-Wallis test. Comparison between categorical data was made using Fisher's exact-test. Statistical power is expressed in P (two-sided; alpha 0.05). Correlations between variables were assessed using Pearson's and Spearman's correlation analyses. Strength and direction of linear relationships are expressed in *r* (*P*-value). Normality was assessed using the Shapiro-Wilk test. Odds ratios were computed using the Baptista-Pike method. Survival was measured from the date of operation to the date of death or date of last follow-up. The prognostic value of the tPMA z-score was analyzed on a receiver operating characteristic (ROC) curve, and optimal cut-offs were evaluated using the Youden index. Survival curves were created using the Kaplan-Meier method. Simple and multiple regression analysis was performed using Cox proportional hazards regression model for 5-year mortality. Multiple regression analysis included all explanatory variables with *P* < 0.1 in the simple regression analysis. Data was analyzed and figures were generated using GraphPad Prism version 8.0.0 for Mac OS X, GraphPad Software, La Jolla, California and IBM SPSS for Macintosh, version 25.0 (IBM Corp., Armonk, N.Y. USA).

## Results

### Study Population and Descriptive Statistics

One hundred sixty-four children had a workup for NB at our institute between October 1, 2008 and January 1, 2016, of which 101 fulfilled inclusion criteria. A total of 63 children were excluded due to unavailable CT or MRI image (*n* = 20), age at CT or MRI image (under 1 year or over 16 years of age) (*n* = 14), incomplete imaging without capturing lumbar levels L3-5 (*n* = 20), poor image quality (*n* = 4), no surgical procedure (*n* = 4), or vertebral anomalies (*n* = 1). Clinical data and imaging of 101 children, of which 47 were girls (47%), with a mean age of 3.92 years (SD 2.71 years) were analyzed. Half of the children had tumors staged INSS 4 (50%), 21 children (21%) had tumors showing a MYCN amplification (MYCN+), and 56 children (55%) had NB with a primary localization in the adrenal gland. The mean WFA z-score was −0.37 (SD 1.11).

Pre-operatively, 32 patients were treated according to the NB2004 High Risk (NB2004-HR) protocol (standard or experimental arm) and 14 patients were treated according to the NB2004 protocol for patients with medium risk as defined by the Society for Pediatric Oncology/Hematology [Gesellschaft für Pädiatrische Onkologie und Hämatologie, GPOH (27, 28)]. Four patients were in the observation group and received one to four cycles of N4 according to NB2004 trial protocol prior to surgery after localized progression or threatening symptoms (27). Seventeen patients did not receive chemotherapy or any other treatment prior to surgery. The remaining patients received mixed protocols, protocols from other studies, or other treatment besides chemotherapy prior to surgery. Additional patient and disease characteristics can be found in Table 1.

**Table 1:** Comparison of pre-, intra-, and short-term post-operative data with sarcopenic status in whole cohort.

	**All study participants (*N* = 101)**	**Sarcopenic (*n* = 64)**	**Non-sarcopenic (*n* = 37)**	***P^a^***
**Patient characteristics**				
Female (%)	47 (47)	36 (56)	11 (30)	0.01^*^
Age at imaging, median (IQR), mo	35 (27, 60)	32 (26, 57)	36 (28, 64)	0.56
Age at diagnosis, median (IQR), mo	26 (14, 45)	26 (16, 46)	27 (12, 46)	0.85
Weight-for-age, mean (SD), *z*	−0.37 (1.11)	−0.80 (1.02)	0.36 (0.85)	<0.001^*^
Height-for-age, mean (SD), *z*	−0.20 (1.25)	−0.58 (1.24)	0.44 (1.01)	<0.001^*^
BMI, mean (SD), *z*	−0.36 (1.23)	−0.63 (1.23)	0.11 (1.04)	0.003^*^
**Disease characteristics**				
Histology, no. (%)				n/a^b^
Ganglioneuroma	10 (10)	1 (2)	9 (24)	
Ganglioneuroblastoma				
Intermixed	5 (5)	4 (6)	1 (3)	
Nodular	7 (7)	4 (6)	3 (8)	
Neuroblastoma, differentiating	23 (23)	17 (27)	6 (16)	
Neuroblastoma, poorly differentiated	11 (11)	7 (11)	4 (11)	
Neuroblastoma, undifferentiated	7 (7)	4 (6)	3 (8)	
More than one classification	5 (5)	3 (5)	2 (5)	
Adrenal gland location, no. (%)	56 (55)	36 (56)	20 (54)	0.84
INSS 4, no. (%)	51 (50)	37 (58)	14 (38)	0.07
*MYCN*+, no. (%)	21 (21) *N* = 100	17 (27) *n* = 64	4 (11) *n*= 36	0.08
**Cancer treatment**				
Pre-operative chemotherapy, no. (%)	*N* = 63	*n* = 40	*n* = 23	0.433
No chemotherapy	17 (30)	9 (22.5)	8 (35)	
NB2004 medium-risk group^c^	12 (19)	8 (20)	4 (17)	
NB2004 high-risk group^c^	34 (54)	23 (57.5)	11 (48)	
**Short-term outcome**				
Time in hospital after surgery, median (IQR), d	12 (9, 23)	14 (10, 24)	11 (8, 21)	0.22
Time in ICU after surgery, median (IQR), d	4 (2, 6) *N* = 99	5 (2, 6) *n* = 64	3 (1, 6) *n* = 35	0.13
Sepsis	3 (3)	2 (3)	1 (3)	>0.99

* *Represents a significant result with P <0.05*.

a *P-value for comparison between sarcopenic and non-sarcopenic*.

b *Statistic cannot be completed due to small number of patients per group*.

c *According to GPOH recommendations (29)*.

*BM I, body mass index; d, days; ICU, intensive care unit; INSS, international Neuroblastoma Staging System; IQR, interquartile range; mo, months; SD, standard deviation: z, z-score*.

Of the 101 children for whom pre-operative tPMA was measured, 21 children were lost to follow-up after being discharged from our hospital and were not included in relapse and survival results. Median follow-up time for the remaining 80 patients was 42 months (IQR 10–77 months). Twenty-three children died at a median of 12 months (IQR 5–40 months) post-treatment. **Figure 4** demonstrates Kaplan Meier survival curves, with panel C demonstrating a significantly higher survival rate after surgery for non-sarcopenic children (Log-rank test, *P* = 0.039).

### Total Psoas Muscle Area

Images were taken at a median of 7 days prior to surgery (IQR 2–17.5 days) and 7 months after first diagnosis of NB (IQR 4–12 months). Forty-five images were CT images (45%) and 56 were MRI images (55%). There was no significant difference in tPMA z-score between CT and MRI images at levels L3-4 (*P* = 0.41) and L4-5 (*P* = 0.16). The correlation between left and right PMA was *r* = 0.97 (95% CI 0.95–0.98; *P* < 0.001) at L3-4 and *r* = 0.97 (95% CI 0.96–0.98; *P* < 0.001) at L4-5, showing that there was no major difference in psoas muscle size between the left and right side at both intervertebral levels. When plotting the absolute values of the tPMA at L3-4 vs. those at L4-5, there was a strong linear relationship (*r* = 0.84; 95% CI 0.76–0.89; *P* < 0.001). On average, the tPMA was 16% larger at L4-5 compared to L3-4. Since correlation of tPMA between both levels was excellent and the psoas muscle was larger and easier to depict at L4-5, further analysis was done using tPMA at lumbar level L4-5 (10).

### Total Psoas Muscle Area and Clinical Data

**Figure 1:**
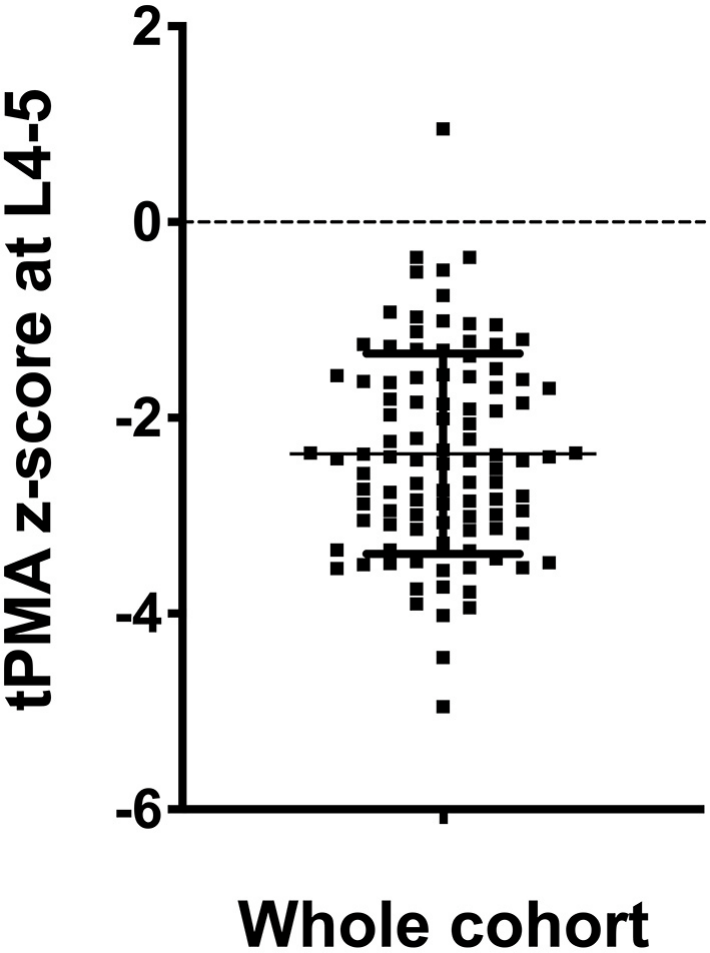
Distribution of tPMA at L4-5. The mean tPMA z-score was −2.37 with a standard deviation of 1.02.

The mean tPMA z-score at L4-5 was −2.37 (SD 1.02) (Figure 1). Children with tumors staged INSS 1 had a higher mean tPMA z-score of −1.03 (SD 0.62) compared to those with tumors staged INSS 3 [mean (SD) −2.40 (1.00); *P* = 0.02], or INSS 4 [mean (SD) −2.58 (0.89); *P* = 0.003]. Girls had significantly lower mean tPMA z-scores compared to boys [mean (SD), −2.72 (1.11) vs. −2.06 (0.84), respectively] (*P* < 0.001). There was no significant association between sex and clinical parameters. Sixty-four children (63%) were sarcopenic, of which 56% were female.

tPMA z-scores at L4-5 correlated moderately with WFA z-score (*r* = 0.54; 95% CI 0.38–0.66; *P* < 0.001) and poorly with HFA (*r* = 0.35; 95% CI, 0.17–0.52; *P* = 0.001) and BMI (*r* = 0.38; 95% CI 0.20, 0.54; *P* = 0.001) z-scores. According to WHO definitions, seven children (7%) were malnourished and seven children (7%) were stunted. Nine children (9%) had a BMI z-score below −2.

**Figure 2:**
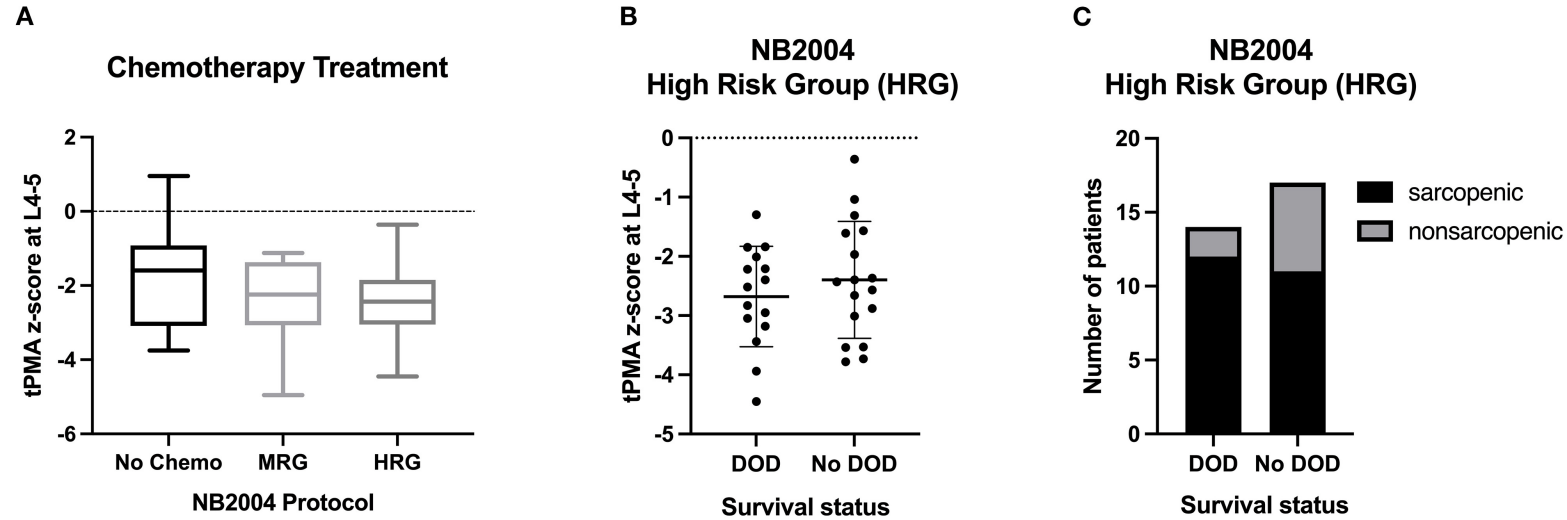
**(A)** Chemotherapy treatment according to NB2004 protocols. Middle line - median; Interquartile range - box; 25–75% - Whiskers; Tukey Test. **(B)** Distribution of the tPMA z-score at L4-5 among survival groups within the NB2004 high-risk group. Middle line - mean; Upper and lower lines - SD. **(C)** Prevalence of sarcopenia among survival groups within the NB2004 high-risk group. Chemo, chemotherapy; MRG, medium risk group; HRG, high-risk group; DOD, death of disease.

Children who received no chemotherapy before surgery had mean tPMA z-scores of −1.94 (SD 1.28) as opposed to those who received either chemotherapy according to the NB2004 medium risk protocol [mean (SD) −2.33 (1.12); *P* = 0.38] or according to the NB2004-HR protocol prior to surgery [mean (SD) −2.53 (0.92); *P* = 0.07]. See Figure 2 for more information.

### Post-operative Short-Term Outcome and Sarcopenia

Three children (3%) experienced a post-operative septic shock, median time in the ICU was 4 days (IQR 2–6 days), and mean total time in the hospital after surgery was 12 days (IQR 9–23 days). Sarcopenia was not associated with these short-term outcome parameters (Table 1).

### Post-operative Long-Term Outcome and Sarcopenia

#### Five-Year Survival and Sarcopenia

**Figure 3:**
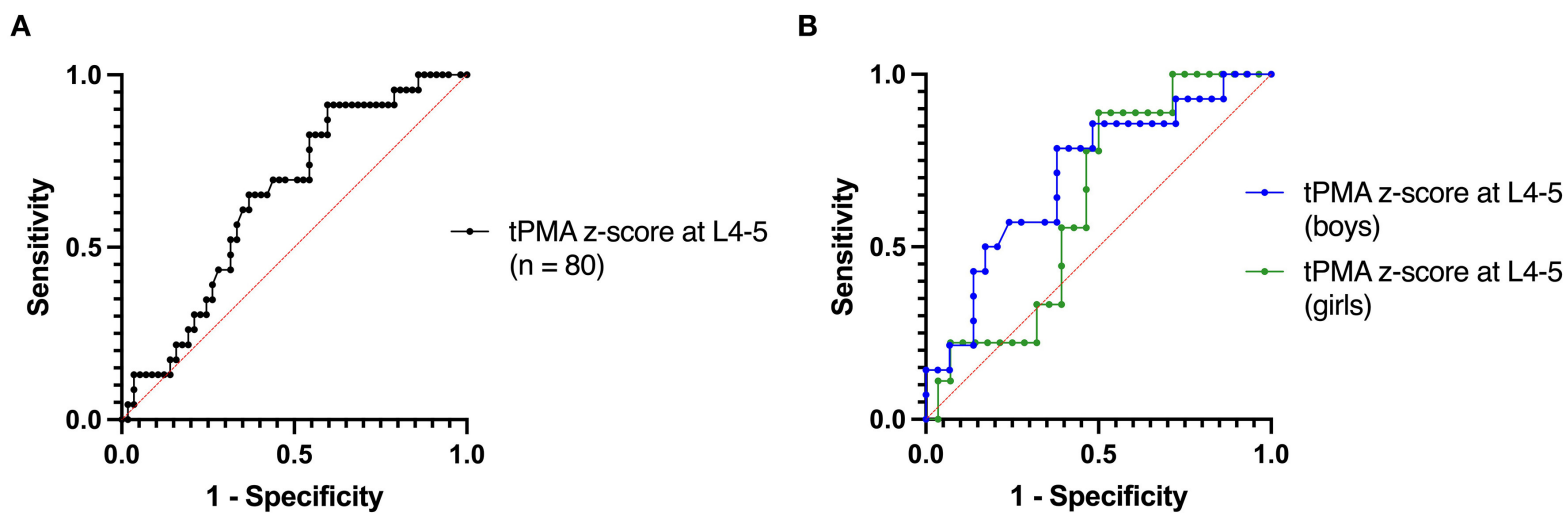
ROC curves for tPMA z-score at L4-5 using 5-year survival **(A)** for all patients with post-operative follow-up and **(B)** according to sex.

Follow-up data was available for 80 patients. Median tPMA was −2.36 (SD 1.04). Of these, 29 children had a relapse (36%), and 23 children died (29%). Sarcopenia was not associated with relapse (Table 2). Figure 3 demonstrates Receiver Operating Characteristic (ROC) curves for tPMA and probability of 5-year survival with an area under the curve (AUC) of 0.65 (95% CI 0.52–0.77; *P* = 0.042) in the overall cohort. Sarcopenia had a sensitivity of 0.82 (95% CI 0.62–0.93) and a specificity of 0.48 (95% CI 0.36–0.61) in predicting 5-year survival. Because girls had significantly lower tPMA z-scores compared to boys, we also stratified the ROC curves according to sex. In girls, the AUC was 0.63 (95% CI 0.44–0.81; *P* = 0.257) and sarcopenia had a sensitivity of 0.89 (95% CI 0.57–0.99) and a specificity of 0.29 (95% CI 0.15–0.47) in predicting 5-year survival (*P* = 0.403). In boys, the AUC was 0.71 (95% CI 0.54–0.87; *P* = 0.029) and sarcopenia had a sensitivity of 0.79 (95% CI, 0.52–0.92) and a specificity of 0.62 (95% CI, 0.44–0.77) (*P* = 0.021) (Figure 3).

**Table 2:** Comparison of long-term post-operative outcome with sarcopenic status in patients with follow-up.

	**Patients with follow-up (*N* = 80)**	**Sarcopenic (*n* = 50)**	**Non-sarcopenic (*n* = 30)**	***P^a^***
**Long-term outcome**				
Relapse, no. (%)	29 (36)	21 (42)	8 (27)	0.23
**Disease status, no. (%)**				
CR	31 (38.75)	11 (22)	20 (67)	
AWD	20 (25)	17 (34)	3 (10)	n/a^b^
PD	6 (7.5)	3 (6)	3 (10)	
DOD	23 (28.75)	19 (38)	4 (13)	

a *P-value for comparison between sarcopenic and non-sarcopenic*.

b *Statistic cannot be completed due to small number of patients per group*.

*CR, complete remission, AWD, alive with disease; PD, progressive disease; DOD, death of disease*.

**Figure 4:**
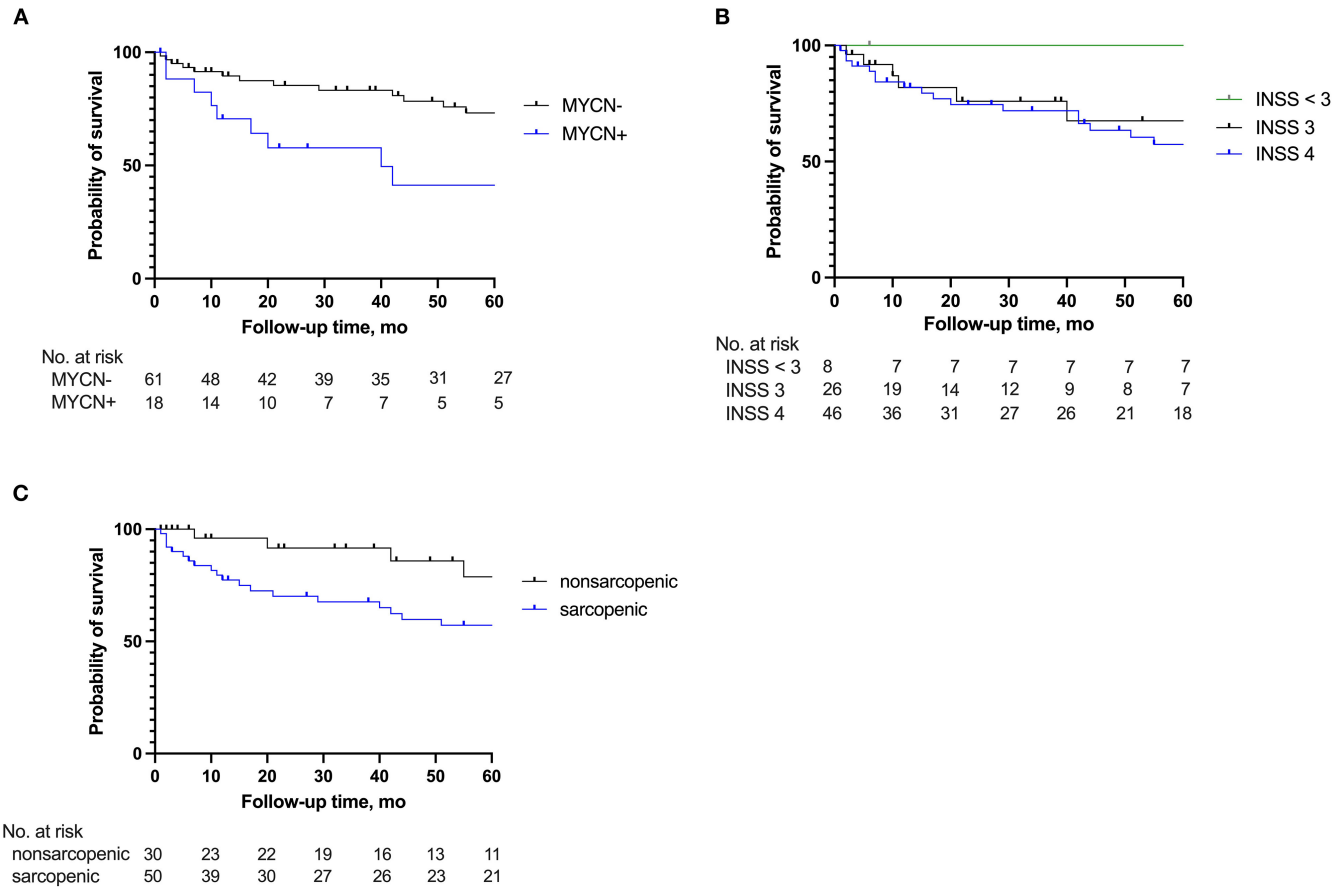
Survival curves 5 years after surgery according to **(A)** MYCN amplification (MYCN+), **(B)** International Neuroblastoma Staging System (INSS) classification, and **(C)** sarcopenic status.

In Figure 4, survival rates are shown. Using a simple regression analysis, sarcopenia, unfavorable tumor histology, MYCN+, and NB2004-HR chemotherapy were significant risk factors for 5-year mortality. Children receiving NB2004-HR were more likely to have a MYCN amplification and be categorized as INSS4. In a multiple regression analysis, pre-operative sarcopenia, age at diagnosis, unfavorable tumor histology, and NB2004-HR chemotherapy remained significant risk factors (Table 3).

**Table 3:** Analysis of pre-operative risk factors influencing 5-year mortality.

	**Simple regression**		**Multivariable regression**	
**Explanatory variable**	***P*-value**	**HR (95% CI)**	***P-*value**	**HR (95% CI)**
Age at diagnosis	0.052	1.01 (1.00–1.02)	0.015^*^	1.01 (1.00–1.03)
Unfavorable tumor histology ^a^	0.025^*^	2.49 (1.09–5.68)	0.043^*^	2.39 (1.03–5.54)
Tumor in adrenal gland	0.08	2.29 (0.90–5.82)		
Sex	0.59	1.26 (0.55–2.92)		
INSS 4	0.16	1.96 (0.77–4.96)		
*MYCN* +	0.006^*^	3.19 (1.39–7.32)		
WFA *z*	0.07	0.71 (0.49–1.02)		
HFA *z*	0.19	0.81 (0.59–1.11)		
BMI *z*	0.08	0.75 (0.54–1.03)		
Sarcopenia	0.039^*^	4.26 (1.00–8.65)	0.048^*^	4.18 (1.01–17.26)
NB2004-HR chemotherapy^b^	0.020^*^	2.77 (1.17–6.54)	0.046^*^	2.46 (1.02–5.92)

* *Represents a significant result with P <0.05*.

a *Includes poorly differentiated neuroblastoma, undifferentiated neuroblastoma, and nodular ganglioneuroblastoma (25)*.

b *According to GPOH recommendations (29)*.

*BMI, body mass index; CI, confidence interval; HFA, height-for-age; HR, hazard ratio; INSS, International Neuroblastoma Staging System; WFA, weight-for-age; z, z-score*.

#### Sub-analysis of Long-Term Outcome and Sarcopenia

During clinical follow-up, children were noted to have a complete remission (CR), to be alive with disease (AWD), to have progressive disease (PD), or to be dead of disease (DOD). Children with a CR had significantly higher pre-operative mean tPMA z-scores of −1.84 (SD, 1.08) compared to children AWD [mean (SD) −2.78 (0.97); *P* = 0.003] and DOD [mean (SD) −2.74 (0.82); *P* = 0.002]. When stratifying according to sex, this difference was especially true in boys (Supplementary Figure 1). Children who were non-sarcopenic prior to surgery had a high likelihood of having a post-operative complete remission (OR, 7.09; 95% CI 2.64–19.82; *P* < 0.001; Sensitivity 0.80, 95% 0.66–0.89; Specificity, 0.65, 95% CI 0.47–079). Supplementary Figure 2 demonstrates that in boys, a normal psoas muscle area is associated with a favorable post-operative prognosis, with non-sarcopenic boys being 23 times more likely to experience a complete remission (95% CI 4.06–114; *P* < 0.001). Similar results were not seen in girls. See Supplementary Figure 3 for distribution of sarcopenia among high-risk pre-operative clinical groups.

## Discussion

In this study, we demonstrate that most children with NB have a small age- and gender-specific tPMA z-score prior to surgery and that sarcopenia was a significant risk factor for poor 5-year survival after tumor surgery. When comparing pre-operative risk factors, pre-operative sarcopenia, as well as age at diagnosis, unfavorable tumor histology, and NB2004-HR chemotherapy were associated with lower 5-year survival rates. In contrast, non-sarcopenic children with NB had higher odds for a complete remission.

Our findings support the results of Kawakubo et al., who also used PMA to assess for sarcopenia in thirteen children with high-risk NB (4). While a different risk stratification was used and the change of PMA was observed, Kawakubo et al. determined that an increase in PMA at lumbar level L3 of >1 cm^2^ post-treatment showed a prolonged overall and progression-free survival (4). In comparison, in our study, we examined the tPMA at lumbar level L3-4 and L4-5 in a much larger cohort mainly treated according to GPOH recommendations (29), allowing for more detailed analyses but nevertheless confirming these results.

A small number of studies have applied the concept of sarcopenia to children with cancer, although definitions and methods vary. Rayar et al. examined the change in skeletal muscle mass, determined using appendicular lean mass on dual-energy X-ray absorptiometry (DXA) scans, during treatment for acute lymphoblastic leukemia (ALL) and found that a decrease in skeletal muscle mass during early therapy was associated with the length of hospital stay (30). Orgel et al. showed that children undergoing treatment for ALL frequently develop a phenomenon called sarcopenic obesity, resulting in a poor correlation between body fat and BMI, measured by DXA (31). For the same cancer, Suzuki et al. used the tPMA at L3 on CT images pre- and post-induction therapy to assess for sarcopenia, and revealed a loss of tPMA after treatment was a predictor of unfavorable events, such as invasive fungal infections (3). Using CT and MRI scans to assess for sarcopenia in children with NB is reasonable because these scans are already part of the pre-operative workup, and tPMA measurements are easy, fast and reproduceable (20, 22). Further, age- and sex-specific reference values are available and easy to calculate using a freely available online tool (10).

Currently, no standardized method exists to diagnose sarcopenia. As a result, researchers have elected to use a range of various imaging modalities and measurement techniques as diagnostic indicators. When comparing imaging modalities, studies have shown that a strong correlation exists between skeletal muscle measurements on CT and MRI images (32–34). Frequently used markers to quantify muscle mass include the total psoas muscle area or index (PMA or PMI) and the skeletal muscle area or index (SMA or SMI) (35, 36). Similar to the BMI adjusting body mass for height, some studies on sarcopenia have adjusted the skeletal muscle area or psoas muscle area of cross-sectional imaging for height. No consensus exists whether adjusting for height yields more accurate results. Our study showed that, using the freehand tool, there is no major difference in PMA between the left and right psoas muscle on both lumbar heights L3-4 and L4-5 in children with NB (*r* = 0.97–0.99). In addition, compared to other imaging modalities and techniques, measuring the tPMA z-score in children with NB has the added benefit of requiring no additional testing or radiation exposure.

Our study suggests that a tPMA z-score below -2, reflecting sarcopenia, could be a useful additional pre-operative biomarker to predict survival in children with NB. The tPMA z-score was independent of all pre-operative factors except INSS status and sex, with girls and children with INSS 3 and INSS 4 having lower tPMA z-scores. A multiple regression analysis did not show sex or INSS status to predict 5-year survival. When taking all pre-operative factors into account, multiple regression analysis showed pre-operative sarcopenia, age at diagnosis, unfavorable tumor histology, and NB2004-HR chemotherapy as predictors of poor outcome.

The association between sarcopenia and poor outcome has been observed in both adults and children with cancer. In short, factors that contribute to sarcopenia in cancer patients include 1) a lack of physical exercise, decreasing protein synthesis through the mTOR and MAPK pathways and increasing protein degradation through tumor necrosis factor (TNF)-alpha and NF-kB (37), 2) endocrine processes, such as the release of stress hormones (38), ghrelin resistance (39, 40), and vitamin D deficiencies (41, 42), seen in some cancer patients, 3) tumor metabolism, which consumes glucose and protein for growth (5, 40, 43, 44), and 4) systemic inflammation reflected by increased levels of inflammatory markers such as IL6 (45). Unfortunately, we were unable to test these hypotheses, but suggest taking these into account for future prospective studies.

Another possible contributor to sarcopenia is chemotherapy. It is speculated that low muscle mass and malnutrition could be explained in part by chemotherapy agents such as cisplatin, doxorubicin, and etoposide (all used in NB2004 protocols), which directly activate NF-kB, causing muscle degradation (6) and chemotherapy side effects, such as diarrhea, vomiting, and mucositis, limiting food intake and increasing fluid loss (5). A recent paper published by Nakamura et al. found that a significant reduction in the psoas muscle index can be observed in the early course of treatment with the JN-H-07 chemotherapy protocol for children with high-risk NB (46). Due to the differences in chemotherapy medications administered and differences in calculating muscle mass (PMA vs. PMI), it is uncertain whether similar changes in PMA would be seen in the patients with high-risk NB in our study. Our study did, however, show that no significant difference in pre-operative tPMA z-scores was observed when comparing children who received chemotherapy according to NB2004 trial protocols and children with no treatment prior to surgery. While Nakamura et al. show male sex to be a predictive factor of PMI recovery, we additionally found that boys were more likely to have higher tPMA z-scores and boys without sarcopenia were more likely to have a favorable post-operative outcome.

It is possible that sarcopenia can be used to identify and direct resources to children early on who are less likely to have a favorable prognosis. Battaglini et al. postulate that exercise training up-regulates anti-inflammatory cytokines, thus inhibiting sarcopenia and anorexia (47). Yazdani et al. show exercise in mice injected with colorectal adenocarcinoma cells protects the liver from injury and metastases; postulating exercise could be beneficial prior to curative surgical treatment by reducing liver pre-metastatic niches (48). While Ballarò et al. agree that exercise has beneficial effects, practicing exercise in the late stages of cachexia could reduce lifespan. Therefore, exercise routines for cancer patients should be carefully evaluated (49). Specific therapies (e.g., megestrol acetate, anabolic steroids, and ghrelin and ghrelin agonists) to increase muscle mass and inhibit cytokine-induced catabolism have also been investigated (50). Unlike in adults, the side effects on a developing child must be considered (5).

In addition, establishing nutritional guidelines (e.g., on protein intake) is crucial in optimizing the nutritional status in this patient population (51). Whether this will improve outcome in children with NB must be determined in future studies, considering a larger muscle mass does not necessarily signify a better muscle function (52). As pointed out by Isiklar et al., according to the definition of sarcopenia in adults by the 2018 statement of the European Working Group on Sarcopenia in Older People (EWGSOP), muscle strength might be a better marker for adverse outcome in the elderly, but this is not known for children (53, 54). Especially in children younger than 5–6 years of age, measurement of strength is challenging due to compliance and missing reference values for traditional tests (e.g., grip strength and 6 min walking distance) (55–57). Nevertheless, adding such a functional evaluation to the analysis of sarcopenia as described here could bear great value, even in children and even if limited. This is especially true when considering that some researchers have recently questioned the premise of the psoas muscle as a suitable sentinel muscle for sarcopenia (58, 59). On the contrary, there is emerging evidence for the use of the tPMA to determine pediatric sarcopenia as a risk factor for poor outcome in solid organ transplantation, hematologic disorders, or acute appendicitis (3, 19, 21, 57, 60). Three complementary pediatric reference values for the tPMA are currently available (10, 61, 62).

Limitations of our study include its retrospective design and the post-operative loss to follow-up of 21 patients. Anthropometric data and clinical pre-, intra-, and post-operative data were not available for all subjects. As mentioned above, neither markers of muscle strength nor other (potentially more precise) nutritional measures, such as the mid-upper arm circumference (MUAC) or triceps skinfold thickness (TCFS), were available. On the contrary, a recent study in children with biliary atresia showed a poor correlation between MUAC and tPMA, supporting the additional value of a tPMA analysis (63).

In conclusion, this study demonstrates that the majority of children with NB had low tPMA z-scores prior to surgery. Furthermore, sarcopenia is associated with reduced long-term survival after tumor surgery. Implications of these findings on the pathophysiology and treatment options for children with NB should be explored in larger prospective cohort studies.

## Data Availability Statement

The original contributions presented in the study are included in the article/Supplementary Material, further inquiries can be directed to the corresponding author.

## Ethics Statement

The studies involving human participants were reviewed and approved by Ethikkommission bei der LMU München. Written informed consent from the participants' legal guardian/next of kin was not required to participate in this study in accordance with the national legislation and the institutional requirements.

## Author Contributions

MB, EL, and AF-P conceptualized the study. AR and JL-Z performed measurements. AR, AF-P, JK, and VV gathered pre-clinical and outcome data. AR, MB, and EL analyzed data, conceptualized manuscript, and wrote manuscript. AR, AF-P, JK, VV, JH, JL-Z, DvS, BH, IS, RK, EL, and MB interpreted data and critically reviewed manuscript. All authors contributed to the article and approved the submitted version.

## Conflict of Interest

The authors declare that the research was conducted in the absence of any commercial or financial relationships that could be construed as a potential conflict of interest.

## Publisher's Note

All claims expressed in this article are solely those of the authors and do not necessarily represent those of their affiliated organizations, or those of the publisher, the editors and the reviewers. Any product that may be evaluated in this article, or claim that may be made by its manufacturer, is not guaranteed or endorsed by the publisher.

## Supplementary Material

Supplementary Figure 1 Distribution of total psoas muscle z-score at L4-5 according to post-operative disease status. Most children with a post-operative complete remission (CR) did not have pre-operative sarcopenia, while the majority of children alive with disease (AWD), a progressive disease (PD), and death of disease (DOD) were sarcopenic. A significant difference was observed between children with a complete remission and children alive with disease (AWD) and dead of disease (DOD) (^*^*P* = 0.006 and ^**^*P* = 0.006, respectively). Middle line - median; Interquartile range—box; 25–75% - Whiskers; Tukey Test.

Supplementary Figure 2 Distribution of sarcopenia according to post-operative disease status **(A)** for all patients with post-operative follow-up, **(B)** for boys, and **(C)** for girls. In boys, a normal psoas muscle area is associated with a favorable post-operative prognosis, with non-sarcopenic boys being 23 times more likely to experience a complete remission (95% CI 0.001).

Supplementary Figure 3 Distribution of sarcopenia according to post-operative disease status in **(A)** patients with a MYCN amplification (MYCN+), **(B)** patients with tumors categorized INSS4, **(C)** patients who received pre-operative chemotherapy exclusively according to the NB2004 high-risk group (NB2004 HRG), and **(D)** patients with unfavorable histology (poorly differentiated neuroblastoma, undifferentiated neuroblastoma, and nodular ganglioneuroblastoma) (25). CR, complete remission; AWD, alive with disease; PD, progressive disease; DOD, death of disease.
