# Supplementary figures and images for: Total Psoas Muscle Area as a Marker for Sarcopenia Is Related to Outcome in Children With Neuroblastoma
Source: Front Surg. 2021 Aug 19;8:718184. doi: 10.3389/fsurg.2021.718184 (PMC8418308; doi:10.3389/fsurg.2021.718184)

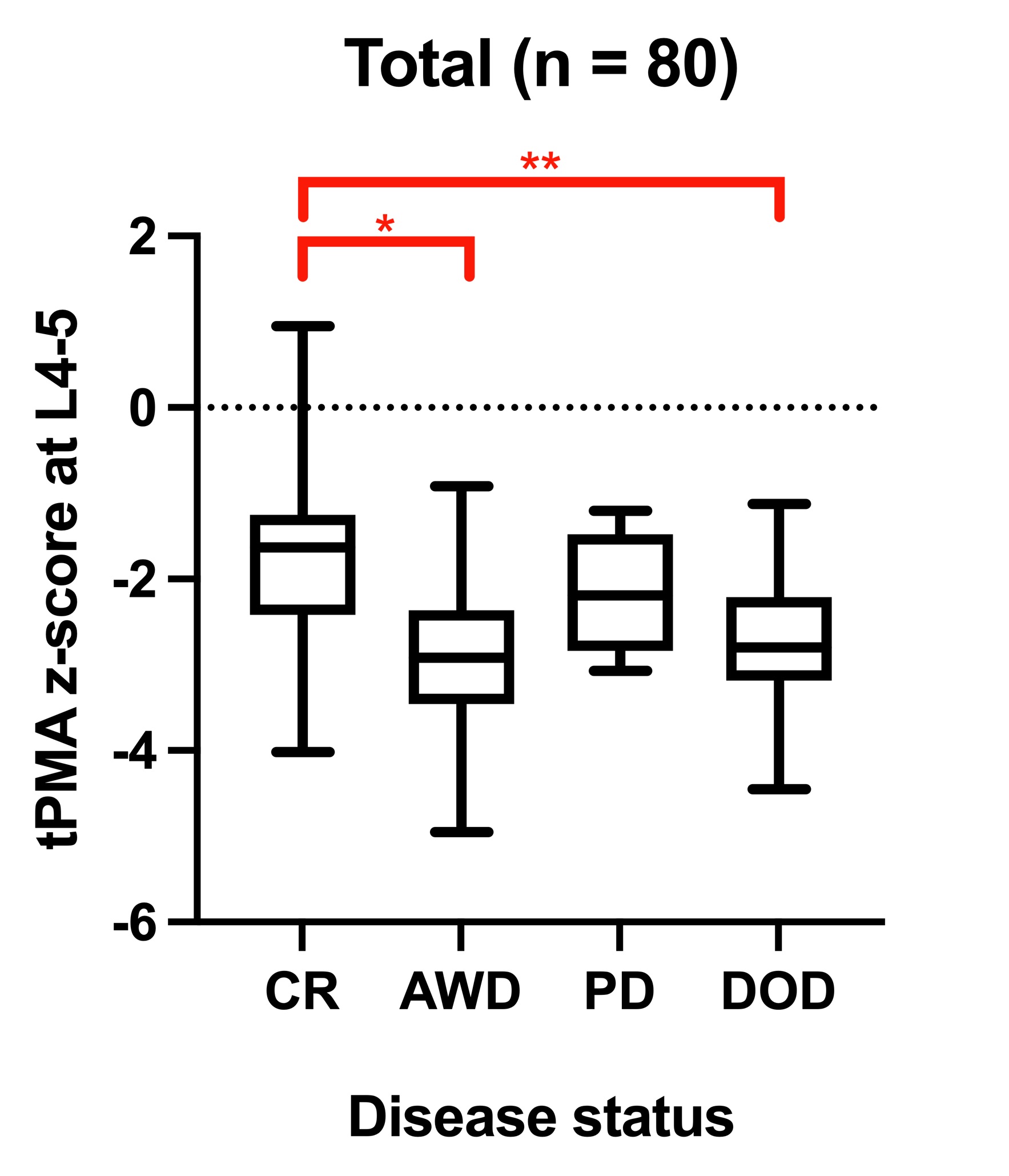

Supplement: Supplementary Figure 1 — Distribution of total psoas muscle z-score at L4-5 according to post-operative disease status. Most children with a post-operative complete remission (CR) did not have pre-operative sarcopenia, while the majority of children alive with disease (AWD), a progressive disease (PD), and death of disease (DOD) were sarcopenic. A significant difference was observed between children with a complete remission and children alive with disease (AWD) and dead of disease (DOD) (*P = 0.006 and **P = 0.006, respectively). Middle line - median; Interquartile range—box; 25–75% - Whiskers; Tukey Test. [file Image_1.JPEG]

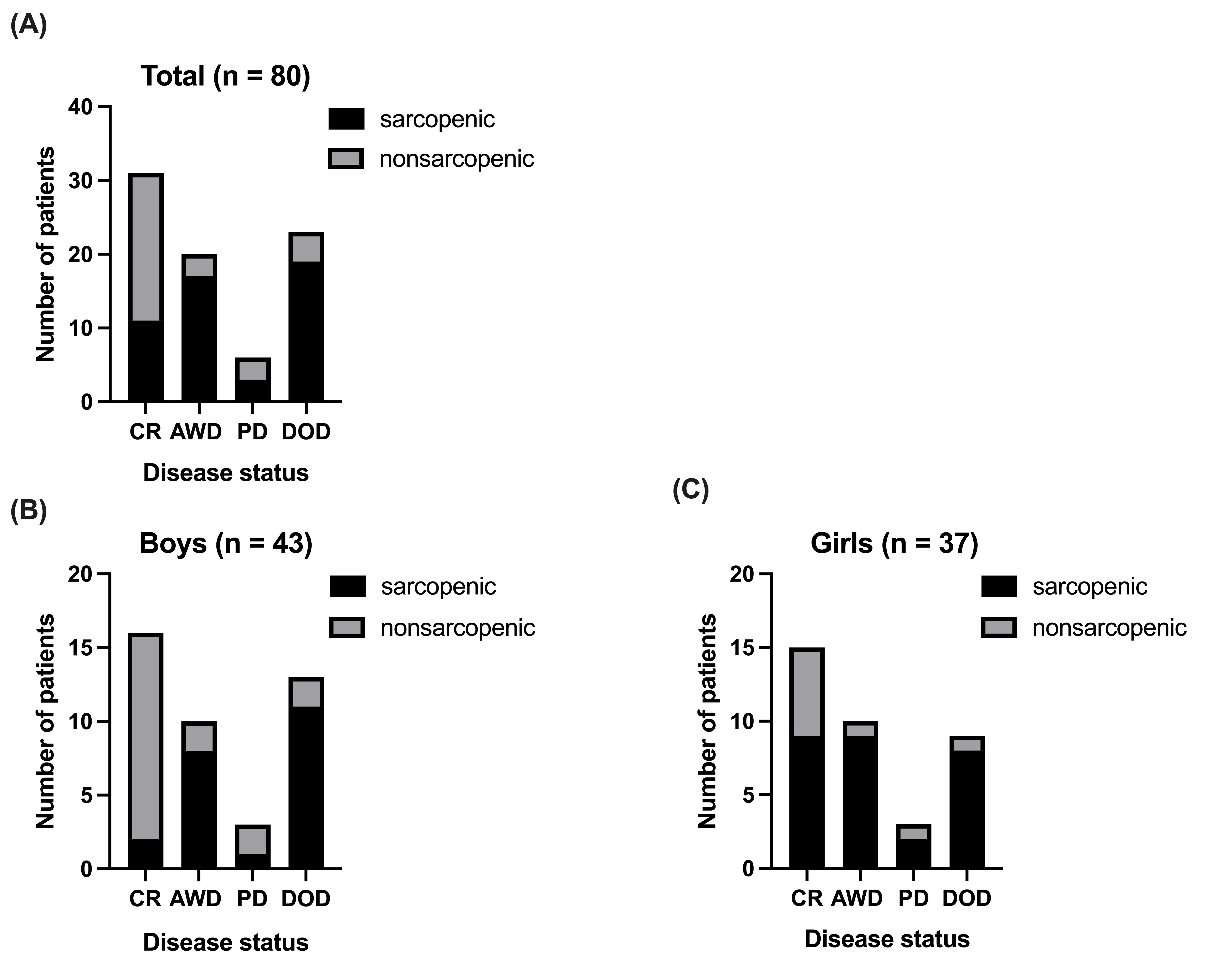

Supplement: Supplementary Figure 2 — Distribution of sarcopenia according to post-operative disease status (A) for all patients with post-operative follow-up, (B) for boys, and (C) for girls. In boys, a normal psoas muscle area is associated with a favorable post-operative prognosis, with non-sarcopenic boys being 23 times more likely to experience a complete remission (95% CI 0.001). [file Image_2.JPEG]

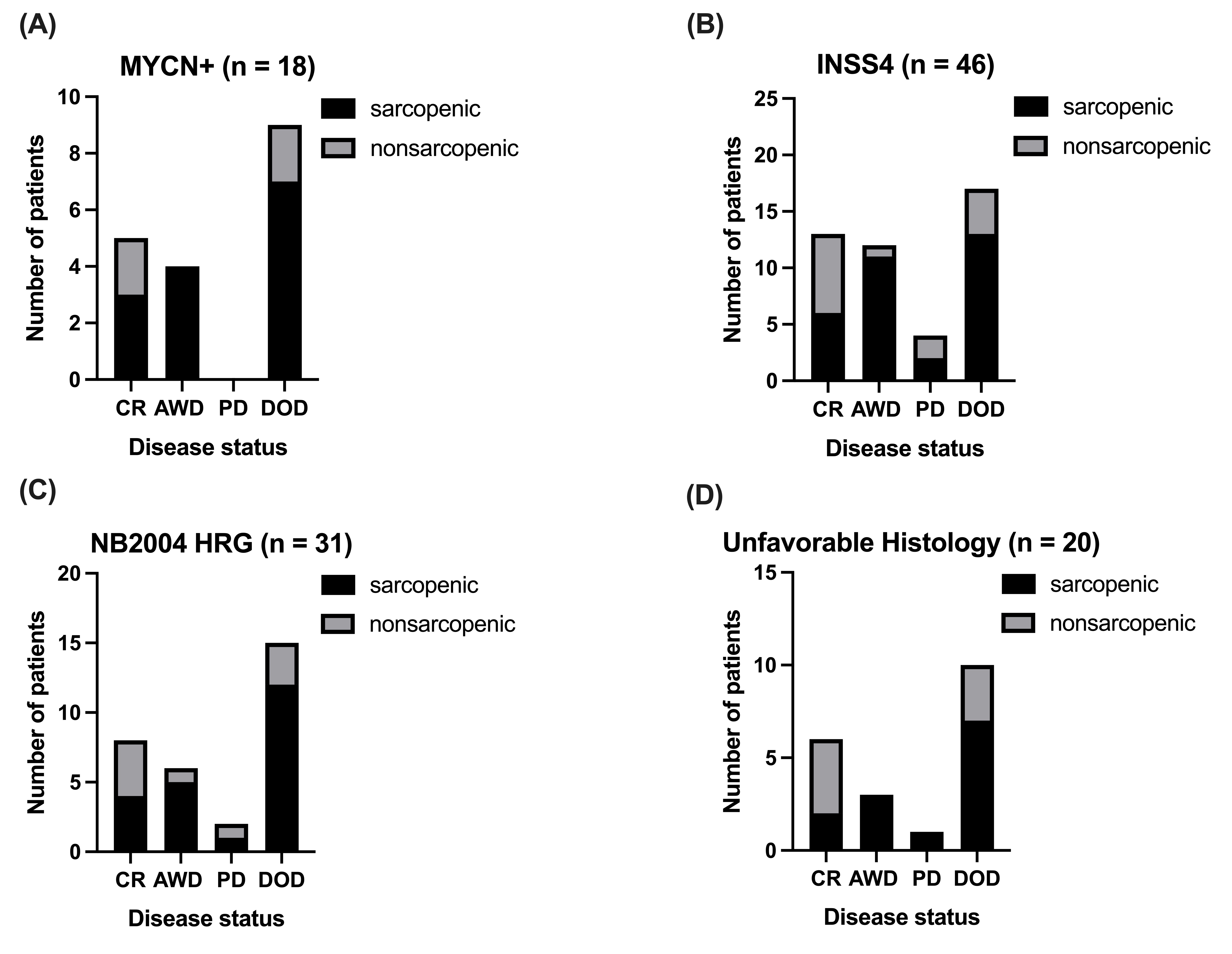

Supplement: Supplementary Figure 3 — Distribution of sarcopenia according to post-operative disease status in (A) patients with a MYCN amplification (MYCN+), (B) patients with tumors categorized INSS4, (C) patients who received pre-operative chemotherapy exclusively according to the NB2004 high-risk group (NB2004 HRG), and (D) patients with unfavorable histology (poorly differentiated neuroblastoma, undifferentiated neuroblastoma, and nodular ganglioneuroblastoma) (25). CR, complete remission; AWD, alive with disease; PD, progressive disease; DOD, death of disease. [file Image_3.JPEG]
